# Health professional-delivered obesity prevention interventions during the first 1,000 days: A systematic review of external validity reporting

**DOI:** 10.12688/hrbopenres.12924.2

**Published:** 2019-10-21

**Authors:** Marita Hennessy, Caroline Heary, Rachel Laws, Luke Van Rhoon, Elaine Toomey, Hazel Wolstenholme, Molly Byrne

**Affiliations:** 1 Health Behaviour Change Research Group, School of Psychology, National University of Ireland, Galway, Galway, Ireland; 2 School of Psychology, National University of Ireland, Galway, Galway, Ireland; 3 Institute for Physical Activity and Nutrition (IPAN), School of Exercise and Nutrition Sciences, Deakin University, Geelong, Victoria, Australia

**Keywords:** External validity, childhood obesity, generalisability, intervention, implementation science, health professional, prevention, replication, systematic review

## Abstract

**Background:** Childhood obesity prevention interventions delivered by health professionals during the first 1,000 days of life show some evidence of effectiveness, particularly in relation to behavioural outcomes. External validity refers to how generalisable interventions are to populations or settings beyond those in the original study. The degree to which external validity elements are reported in such studies is unclear however. This systematic review aimed to determine the extent to which childhood obesity interventions delivered by health professionals during the first 1,000 days report on elements that can be used to inform generalizability across settings and populations.

**Methods:** Eligible studies meeting study inclusion and exclusion criteria were identified through a systematic review of 11 databases and three trial registers. An assessment tool based on the RE-AIM (Reach, Effectiveness, Adoption, Implementation, Maintenance) framework was used to assess the external validity of included studies. It comprised five dimensions: reach and representativeness of individuals, reach and representativeness of settings, implementation and adaptation, outcomes for decision making maintenance and/or institutionalisation. Two authors independently assessed the external validity reporting of 20% of included studies; discrepancies were resolved, and then one completed assessments of the remaining studies.

**Results:** In total, 39 trials involving 46 interventions published between 1999 and 2019 were identified. The majority of studies were randomized controlled trials (n=24). Reporting varied within and between dimensions. External validity elements that were poorly described included: representativeness of individuals and settings, treatment receipt, intervention mechanisms and moderators, cost effectiveness, and intervention sustainability and acceptability.

**Conclusions:** Our review suggests that more emphasis is needed on research designs that consider generalisability, and the reporting of external validity elements in early life childhood obesity prevention interventions. Important gaps in external validity reporting were identified that could facilitate decisions around the translation and scale-up of interventions from research to practice.

## Introduction

In 2018, approximately 41 million children under the age of five years were classified as having overweight or obesity ^1^. Child obesity prevention is a public health priority, with early life intervention advocated ^2^. Effective, scalable, and affordable strategies that do not widen health inequities are needed to address this issue ^3, 4^. In addition, interventions that can be embedded into ongoing practice and existing systems are required, rather than implementing interventions that are resource-intensive and cannot be maintained in the long-term ^5, 6^. This was echoed in a recent research prioritisation study in which ‘Implementation science’ and ‘How to integrate obesity prevention into existing service structures’ were the third and fourth ranked research priorities identified by researchers, policymakers and practitioners ^7^. To date, there has been limited scale-up of childhood obesity prevention interventions.

Appraising scalability prior to investment is vital ^8, 9^. Scalability is defined by the World Health Organization as ‘the deliberate effort to increase the impact of successfully tested health interventions so as to benefit more people and to foster policy and program development on a lasting basis’ ^10^. An understanding of the external validity of the intervention is critical to determining scalability, in addition to understanding the fit between an intervention and the political and strategic context. External validity refers to the generalizability of the results of an intervention to or across target populations or settings, while applicability refers to generalizability to any populations or settings ^11^. To understand the external validity of an intervention, decision makers need to have sufficient information on the reach and acceptability of the intervention, core intervention components required for fidelity, any differential effects on the target population, unintended consequences, costs versus benefits, and the clinical or policy significance of the intervention effects to inform decisions about whether interventions should be scaled-up ^12– 16^. The poor reporting of external validity elements in childhood obesity prevention research also limits decision-makers’ ability to determine the likely scalability of these interventions, limiting the implementation of effective interventions into routine practice ^17, 18^.

As public health interventions are usually complex, and context dependent, it can be difficult to assess their generalisability to other contexts ^14^. There are many tools for assessing generalisability, however, there is no consensus on which should be used, or when ^14^. Indeed, Burchett and colleagues argue that such tools may not be the best method for generalisability assessments, instead advocating a focus on mechanisms of action through which an intervention exerts its effect – and which contextual elements underpin them, rather than solely on intervention characteristics ^14^. To improve reporting across behavioural interventions and enhance the translation of research into practice, Glasgow and colleagues developed the RE-AIM (Reach, Effectiveness, Adoption, Implementation, Maintenance) framework ^19, 20^. Reach is the number, proportion of the intended target population, and the representativeness of participants compared with the intended target population. Effectiveness (or efficacy, depending on the study design) is the degree to which the intervention changes behavioural, quality of life, and participant satisfaction outcomes as well as physiologic endpoints, and includes attention to positive, unintended and negative results. Adoption is the number and proportion of settings and staff members that agree to initiate an intervention and how representative they are of the target setting and staff. Implementation is the degree to which settings and staff members deliver an intervention as intended, the adaptations made, and the related costs. Finally, maintenance is sustained effectiveness at the participant level and sustained (or adapted) delivery at the setting or staff level. At the individual level, it refers to the long-term results of intervention (defined as a minimum of six months following the last contact) ^20– 22^. RE-AIM is the most frequently applied framework in the translation of research evidence into policy and practice ^23^. It has been used to assess reports of external validity factors across a variety of areas, including weight loss maintenance interventions ^24^, behavioural interventions that target physical activity ^25^, mobile health physical activity promotion interventions ^26^, physical activity promotion in Latin American populations ^27^, behavioural intervention studies conducted in community settings ^28^, school health promotion studies ^29^, behaviour change interventions in healthcare settings ^30^, and housing improvement ^31^.

Based on the RE-AIM framework, Green and Glasgow proposed a set of ratings to assess external validity ^21^. These were further adapted by Laws and colleagues ^32^ and have been used to assess external validity in diabetes prevention research ^32^ and obesity prevention in children aged 0–5 years ^18^.

Reviews of external validity reporting in childhood obesity interventions identify insufficient reporting of elements necessary to make decisions about generalisability ^17, 18^. A review of external validity reporting in 19 long-term follow-up childhood obesity prevention trials (children aged 0–18 years) published between 1980 and 2004 found that all studies lacked full reporting on potential generalizability and dissemination elements; the most infrequent were reports of setting level inclusion and exclusion criteria and representativeness, characteristics regarding intervention staff, implementation of intervention content, costs, and program sustainability ^17^. A more recent review of external validity reporting in 32 trials of interventions to prevent obesity or improve obesity related behaviours in children aged 0–5 years from socioeconomically disadvantaged or Indigenous families found similar issues with reporting ^18^. Health professional-delivered interventions to prevent childhood obesity during the first 1,000 days of life (i.e. the period from conception to a child’s second birthday) have limited impacts on adiposity/weight outcomes, but have more positive impacts on behavioural outcomes ^33^. Despite the increasing numbers of trials to assess the impact of early life obesity prevention interventions, there is relatively little reporting on the potential for these interventions to be translated into routine practice. Furthermore, there is little evidence that interventions with demonstrated efficacy have been translated beyond the research setting and been broadly adopted. Given that it can take up to 17 years to translate evidence into practice ^34^, it is important to assess the extent to which trials report on factors that can provide additional explanation for variability in intervention outcomes, insights into successful adaptations of interventions, inform generalizability across settings and populations, and help guide policy decisions.

This study aims to determine the extent to which childhood obesity interventions delivered by health professionals during the first 1,000 days report on factors that can be used inform generalizability across settings and populations, and to provide recommendations for researchers planning to conduct similar studies.

## Methods

We conducted a systematic review of obesity prevention interventions delivered by health professionals targeting children in the first 1,000 days of life. A separate paper reports on the effectiveness of such interventions and what behaviour change theories and/or techniques are associated with more effective intervention outcomes ^33^. The review protocol was registered with the International Prospective Register for Systematic Reviews (PROSPERO) CRD42016050793 on 3 ^rd^ November 2016. This paper adheres to the PRISMA (Preferred Reporting Items for Systematic Reviews and Meta-Analyses) standardised reporting guidelines ^35^; the PRISMA checklist is available on OSF ^36^.

### Search strategy

Key word searches, using combinations of key words and Medical Subject Headings (or equivalent), were used across six concepts using the AND Boolean operator: (1) child; (2) mother/parent; (3) BMI/obesity; (4) nutrition/physical activity/sleep/parenting; (5) intervention/prevention; (6) randomised controlled trial (RCT)/quasi-randomised trials. Within each of the categories, keywords were combined using the “OR” Boolean operator. The search strategy was purposefully broad enough to capture any study which might have assessed weight-related measures in children under the age of two. The search strategy was initially developed in Embase ^®^ (see extended data ^36^), appropriately tailored for use within the other databases, and piloted before final searches were run.

One reviewer (MH) searched the following databases from inception to 04 April 2019 using pre-specified search strategies: CINAHL Complete (EBSCOhost; 1994); Embase ^®^ (Elsevier; 1980); MEDLINE (Ovid ^®^; 1966); PsycINFO (Ovid ^®^; 1978); PubMed (1996); The Cochrane library databases: The Central Register of Controlled Trials; Database of Systematic Reviews; Database of Abstracts of Reviews of Effect (Wiley; 1996). Conference proceedings and other grey literature were searched on: Open Grey (INIST-CNRS; 2011) and Web of Science™ (Thomson Reuters). ProQuest Dissertations & Theses Global, and ProQuest Dissertations & Theses – UK and Ireland, were used to identify eligible dissertation and thesis studies internationally. We also searched the International Clinical Trials Registry Platform Search Portal, ClinicalTrials.gov, and the ISRCTN registry to identify any ongoing or unpublished research trials. Reference lists of previous systematic reviews on this topic were manually searched, as well as those of retrieved full texts.

Supplementary materials and trial registry protocols were also checked. No restrictions were applied to: language; date of publication; the length of follow-up of outcomes (given the diversity reported within systematic reviews to date); type of setting; mode of delivery. Records were de-duplicated in Endnote, imported into COVIDENCE and any remaining duplicates removed.

### Study inclusion and exclusion criteria

Table 1 details the study inclusion and exclusion criteria. We included randomised controlled trials, including cluster-randomised controlled trials, or quasi-randomised trials comparing any behavioural intervention, delivered by health professionals, with ‘usual care’/active comparator which aimed to prevent overweight/obesity in children under the age of two that were born at term. Studies had to report at least one infant/child-related adiposity and/or weight outcome measure at follow-up, which could be immediately post-intervention, or at any time point thereafter); trials only reporting infant birth weight were excluded.

**Table 1.** Study inclusion criteria.

**Design**	Randomised, and quasi-randomised, controlled trials, including individual cluster randomised trials
**Participants**	Studies which targeted pregnant women and/or parents (including mothers/fathers/carers/guardians) of healthy infants less than two years old and/or infants born at term gestation (37 to 42 weeks of gestation) and up to two years of age. ○ No restrictions for sex, ethnicity, socio-economic group, or region, were applied. ○ Studies where children aged under two years were part of a family group receiving the intervention were included only if data could be extracted separately for these children
**Intervention**	○ Behavioural interventions designed to prevent obesity (by directly/explicitly focusing on childhood obesity prevention, or by indirectly focusing on childhood obesity-related risk factors) in infants (e.g. individual counselling, face-to-face sessions, audio-visual packages, support groups, online interventions/forums) delivered by health professionals antenatally and/or up to a child's second birthday. ○ Behavioural interventions were defined as “those that require the active participation of a target group (e.g. patient, individual, health professional) in a programme delivered by a trained interventionist with the goal of changing health- related behaviour” ^37^. ○ Interventions targeting key risk factors for childhood obesity ^38^, including: early rapid weight gain, infant feeding method, timing of introduction of solid foods, and gestational weight gain ○ Health professionals were defined according to the International Standard Classification of Occupations (ISCO) ISCO- 08 ^39^. For the purposes of this review, research nurses, lactation consultants, psychologists, and social workers were also classified as health professionals
**Comparator**	Participants who were not exposed to an intervention/wait-list control, or an active comparator, or who received 'usual care'. ‘Usual care’ is defined as standard support and/or appointments without an obesity prevention focus
**Outcomes**	Primary ○ Infant/child body mass index (BMI) z score ○ Additional anthropometric/growth-related: e.g. growth rates (weight gain, linear growth, and head growth, change in BMI z score), percent fat content, ponderal index, skin-fold thickness Secondary (*intermediate behavioural outcomes) ○ Diet-related*: e.g. breastfeeding initiation and duration (total and exclusive); dietary intake and quality; timing of introduction of solid food(s) ○ Feeding/eating behaviour-related*: e.g. responsive feeding practices ○ Physical activity-related*: e.g. physical activity, tummy time, play, screen time ○ Sedentary time/behaviour-related*: e.g. frequency/time spent: being inactive, doing specific low-energy behaviours such as screen time ○ Sleep* ○ Environment-related*: e.g. outcomes related to the physical (e.g. food availability) and social environment ○ Cost effectiveness/costs of the intervention
**Publications**	Trials reported only as abstracts were deemed eligible for inclusion if sufficient information was available from the report, or from contact with the authors, to fulfil the inclusion criteria

### Study selection

MH and LT independently screened titles and abstracts against the inclusion criteria, and following the retrieval of full-texts, MH and LvR independently reviewed them for inclusion. Disagreements were resolved through discussion, with a third author (MB / CH / RL) where necessary.

### Data extraction

All published papers and supplementary material related to the study (e.g. protocol papers and trial registry protocols, reference to websites with working hyperlinks, long-term follow-up studies) were used alongside the included article for data extraction. Data were extracted by one author (MH) using a pre-piloted data extraction tool (see extended data ^36^), with 20% double-checked by a second reviewer (HCW). Intervention descriptions were extracted following the criteria outlined in the TIDieR reporting guidelines ^40^. The external validity assessment tool previously developed by RL ^18, 32^ was used to assess the extent to which included studies/trials reported on elements that would aid decision-making around whether the findings of such studies/trials could be generalised to populations or settings beyond those in the original study ^21^. This tool includes five main dimensions (defined in Table 2): 1) reach and representativeness (individuals); 2) reach and representativeness (settings); 3) implementation and adaptation (of intervention), which includes fidelity considerations; 4) outcomes for decision makers; 5) maintenance and institutionalisation (i.e. the potential for implementation of the intervention in routine service delivery). Included studies were coded according to whether they met each criterion (yes, no, or not applicable). Initially, two authors (MH and RL) independently assessed the external validity reporting of 20% of included studies. Any discrepancies were resolved through discussion, and then one author (MH) completed assessments of the remaining studies. We did not exclude any studies on the basis of the effectiveness and/or quality assessment.

**Table 2.** Number and percentage of studies reporting external validity elements ^1^.

External validity dimension	Definition	Studies reporting	
		Yes/Total ^2^	%
**Reach and representativeness of individuals**			
Target population for generalizability	Is the intended target population acknowledged/stated (at the individual level) for which the findings intend to be generalised to?	38/39	97
Method to recruit target population	Was information provided about how the target population was recruited/ reached (e.g., radio, newspaper, TV, school meeting)?	30/39	77
Inclusion or exclusion criteria	Were individual inclusion and exclusion criteria stated?	38/39	97
Enrolment rate	Is the enrolment rate or data needed to calculate the enrolment rate among individuals reported? Proportion of people who are eligible for participation who actually enrol in the study	26/39	67
Recruitment rate	Is the recruitment rate or data needed to calculate the recruitment rate among individuals reported? Proportion of potential participants (those invited or expressing interest) who actually enrol in the study	26/39	67
Representativeness of individuals	Are there comparisons between individuals who participated versus either (1) those who declined to participate or (2) target population?	10/39	26
Participant characteristics	Are all of the following reported: •Gender •Age •Any socioeconomic indicators (education, employment status, or income) •Participation by racial or ethnic minority groups	21/39	54
**Reach and representativeness of settings**			
Target setting	Is the target setting for intervention delivery stated (such as workplace, general practice, outpatient facilities, churches, etc.)?	35/38	92
Method to recruit setting	Is information provided about how the site(s) within a given setting were recruited/reached to participate in delivering the intervention?	4/28	14
Inclusion or exclusion criteria	Were inclusion and exclusion criteria for selection of sites within a given setting stated? In the case of single sites, were the characteristics of the site described?	6/28	21
Participation rate	Is the participation level or data need to calculate the participation level among eligible sites reported (only applies to studies with more than one site)?	1/19	5
Representativeness of setting(s)	Are there comparisons between site(s) participating in the intervention and 1) those that decline to participate or 2) the target setting?	1/28	4
**Implementation and adaptation**			
Intervention characteristics	Were the intervention components described?	38/39	97
Intervention adaptation	Is information reported about how the study intervention is similar or different to original efficacy studies? Note: Only applicable to studies where an intervention is adapted from a previous trial	0/5	0
Time to deliver intervention described	Is the number and length of sessions or time required to deliver the intervention described?	24/37	65
Intervention delivery and exposure	Was the extent to which individuals were exposed to the intervention described? (e.g. proportion of planned intervention sessions actually attended (dose); content delivered as specified; provider adherence to intervention plan)	24/37	65
Delivery agents: characteristics and training	Is information provided on who delivered the intervention, such as the type of professional, or the amount of experience, skill or training required to deliver the intervention?	37/39	95
Methods to recruit delivery agents	Is information provided about how the delivery agents were identified/selected?	3/36	8
Delivery agents’ participation	Is the participation level amongst delivery agents reported (% of delivery agents agreeing to participate)?	4/35	11
Fidelity assessment: treatment receipt	Is information reported about whether the program was received as intended? (e.g. degree to which the participants understood the intervention and/or ability to perform the intervention skills)	4/39	10
Mechanisms for intervention effects	Was retrospective analysis conducted to identify the mediating variables through which the intervention achieved its effect?	2/39	5
**Outcomes for decision making**			
Outcomes that can be compared to standards	Are outcomes (at least one) reported in a way that can be compared to either clinical targets or public health goals?	36/39	92
Adverse consequences	Does the article report whether they examined the occurrence of unintended consequences?	18/39	46
Effect moderators by participant characteristics	Are there any analyses of moderator effects by subgroups of participants	10/39	26
Effect moderator by delivery agent/ setting	Are there any analyses of moderator effects by delivery agents or settings	0/37	0
Dose response effect of intervention (sensitivity)	Are there sensitivity analyses to assess dose-response effects of the intervention?	1/39	3
Total costs of intervention	Are total costs of the intervention presented?	6/39	15
Cost of intervention components	If costs are presented, were the costs itemized by intervention components (e.g., personnel, equipment)?	4/6	67
Cost effectiveness	If costs are presented, was there any analysis done to assess cost- effectiveness or cost-benefit of the program or policy?	3/6	50
**Maintenance / institutionalisation**			
Long term effects (at least 12 months) ^3^	Are data reported on longer term effects on health-related outcomes, at least 12 months following program implementation, or environmental or policy change?	19/39	49
Institutionalization: sustainability / plans for sustainability	Are data reported on the sustainability (or reinvention or evolution) or plans for sustainability of the intervention?	4/39	10
Attrition	Are data reported on the number of individuals dropping out and/or lost to follow up	38/39	97
Differential attrition (by condition or population sub-group)	Are data on attrition by condition or population sub-group reported?	35/39	90
Representativeness of completers/ dropouts	Did the study report statistically significant differences in those that dropped out of treatment and those that finished?	19/38	50
Acceptability of the intervention by stakeholders	Was information provided about acceptability of the intervention by stakeholders?	14/39	36

Notes:

^1^Laws *et al*. (adapted from Green *et al*.)

^2^Total = the no. of overall studies (n=39) minus the no. of studies reporting not applicable to the relevant element

^3^In RE-AIM (Reach, Effectiveness, Adoption, Implementation, Maintenance), long-term results of intervention are defined as a minimum of six months following the last contact; long-term is defined as a minimum of 12 months by Laws *et al*.

## Results

**Figure 1.**
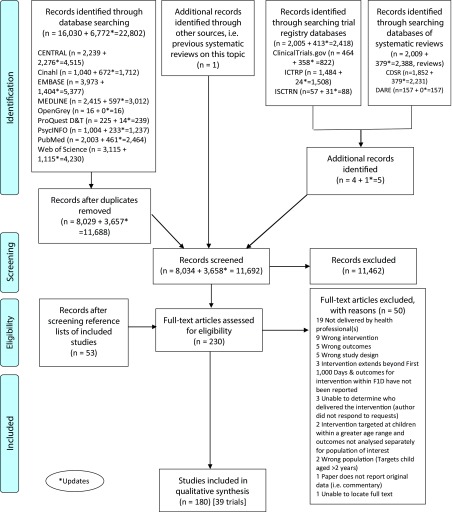
PRISMA flow diagram.

Electronic and hand searches identified 27,609 references (see Figure 1). Following duplicate removal and title and abstract screening, 230 references were selected for full text review. We identified 39 eligible studies with 46 unique intervention arms and a total of 180 eligible papers ^41– 79^. Five trials had more than one eligible intervention arm ^60, 65, 68, 75, 77^.

Studies were mostly published from 2011 onwards (n=34), conducted in high-income countries (n=33), and targeted the period from birth to 2 years only (n=26). They focused on a range of behaviours and outcomes, including: multiple infant behaviours (n=13); infant feeding: formula feeding / breastfeeding / introduction to solids (n=10); maternal diet/physical activity/gestational weight gain (n=9); infant feeding: breastfeeding only (n=8). Only 16 of the 46 interventions were clearly delivered as part of routine care, with a further two partly delivered as such. Details of intervention descriptions and outcomes are available as extended data ^36^.

The assessment of the reporting of external validity elements of the 39 included studies is summarised in Table 2, with a summary by study available as extended data ^36^. Inter-rater reliability, using percent agreement, was high (88.2%). Full details of the these assessments, including supporting statements for each study, are available as extended data ^36^. The number and percentage of studies reporting all elements of each dimension of external validity are outlined in Table 3.

**Table 3.** Number and percentage of studies reporting all elements of each dimension of external validity ^1^.

External validity dimension	No./Total ^2^	%	Studies
Reach and representativeness of participants	6/39	15	NOURISH, PRIMROSE, Baby Milk Trial, INSIGHT, BLISS, POI
Reach and representativeness of settings	9/38	24	ProKind, Baby Milk Trial, Minding the Baby, SLIMTIME, INSIGHT, BLISS, POI, Healthy MOMS, Healthy Beginnings
Implementation and adaptation	0/39	0	None
Outcomes for decision making	0/39	0	None
Maintenance / institutionalisation	1/39	3	INSIGHT

Notes:

^1^Laws *et al*. (adapted from Green *et al*.)

^2^No. taken as sum of no. of studies reporting yes or not applicable to each of the element. Total excludes any studies for which the external validity criterion was not applicable (e.g. Grow2Gether was a social media intervention therefore ‘research and representativeness of settings’ criterion was not applicable).

### Reach and representativeness of participants

Only 15% of studies reported on all elements of this external validity dimension ( Table 3). While almost all studies outlined the target population for generalizability (97%) and inclusion and exclusion criteria (97%), less reported the recruitment method (77%), enrolment rate (67%), and recruitment rate (67%) ( Table 2). Just over half (54%) reported all of the specified participant characteristics - gender, age, any socioeconomic indicators (education, employment status, or income – and participation by racial or ethnic minority groups. Only one in four studies included comparisons between individuals who participated versus either (1) those who declined to participate or (2) target population.

### Reach and representativeness of settings

One in four studies reported on all elements of ‘reach and representativeness of settings’ ( Table 3). Almost all studies provided details of the target setting for intervention delivery (92%); however, the remaining criteria were poorly described: inclusion and exclusion criteria (21%), how settings were recruited/reached to participate in delivering the intervention (14%) ( Table 2). Only one study reported the participation level among eligible sites (5%); this was also the case for the representativeness of setting(s) (4%).

### Implementation and adaptation

No studies reported on all elements of this external validity dimension ( Table 3). Most studies described the intervention characteristics (97%) and the characteristics and training of delivery agents (95%). Less described the time to deliver the intervention (65%), and intervention delivery and exposure (65%) ( Table 2). Delivery agents’ participation (11%), fidelity assessment: treatment receipt (10%), methods to recruit delivery agents (8%), and mechanisms for intervention effects (5%) were very poorly reported. Only five of the studies tested an intervention that was adapted from a previous trial - none reported on how the study intervention was similar or different to original efficacy studies.

### Outcomes for decision making

No studies reported on all elements of ‘outcomes for decision making’ ( Table 3). Almost all studies reported outcomes in a way that could be compared to either clinical targets or public health goals (92%) ( Table 2). Less than half of studies reported whether they examined the occurrence of unintended consequences (46%). Only six studies reported the total costs of the intervention (15%); of these, four studies reported the cost of intervention components (67%), and three examined cost effectiveness (50%). Ten studies (26%) examined effect moderators by participant characteristics; however, none reported effect moderators by delivery agent/setting. Only one study (3%) reported a sensitivity analyses to assess dose-response effects of the intervention.

### Maintenance / institutionalisation

Only one study – INSIGHT – reported on all elements of maintenance / institutionalisation ( Table 3). Almost all studies (97%) reported on the number of individuals dropping out and/or lost to follow up ( Table 2). Data on attrition by condition or population sub-group reported by 90% of studies (Note: we took condition to mean by intervention or control group). Only 50% of studies addressed the representativeness of completers/dropouts. Half of studies (49%) reported data on longer term effects on health-related outcomes (at least 12 months following program implementation, or environmental or policy change). Only 10% of studies reported on the sustainability (or reinvention or evolution) or plans for sustainability of the intervention. Only 36% reported on the acceptability of the intervention by stakeholders.

## Discussion/ Conclusion

Early life interventions delivered by health professionals have the potential to influence important health behaviours, in addition to child weight. Understanding the reporting of external validity elements of such interventions is vital to address their potential for translation and scalability, as well as replication efforts. In this systematic review we identified 39 studies, representing 46 interventions. External validity elements that were generally well reported included target populations and settings, participant inclusion and exclusion criteria, intervention characteristics, delivery agents, outcomes, and attrition. Similar to other reviews of childhood obesity interventions ^17, 18^, however, we identified important gaps in the reporting of external validity elements within studies, and factors that could enhance translation and scale-up of interventions across all five external validity dimensions. External validity elements that were poorly reported included: representativeness of individuals and settings, treatment receipt, intervention mechanisms and moderators, cost effectiveness, and intervention sustainability and acceptability.

Key gaps in informing the translation and scalability of health professional-delivered early life obesity prevention interventions were identified in this review. These included understanding the representativeness of settings, and whether these settings and delivery agents could be engaged to deliver these types of interventions in a sustained way, in a way that is acceptable to those involved. This is especially important given that only 16 of the 46 interventions (35%) in this review were clearly delivered as part of routine care, with a further two partly delivered as such, i.e. contacts as part of routine care but additional contacts also (Starting Early ^78^ and STRIP ^79^). The focus of the majority of studies was on establishing efficacy rather than effectiveness or how such interventions could be scaled up and translated into routine practice. This may account for the poor reporting of external validity in relation to settings and delivery agents. It could be argued that efficacy trials should not be held to the same level of accountability regarding reporting of external validity elements. Such information however is important regardless of trial type, not only to inform generalisability, but also to enhance understanding of the active ingredients of interventions and core components to retain in effectiveness trials or the scale-up of interventions.

Reporting of external validity elements considered important to inform decision makers was generally poor also. This included cost and cost-effectiveness measures, and an understanding of the intervention mechanisms and dose-response effects. While most interventions that are scaled up need to be adapted to fit the delivery context, knowing information about dose-response and the mechanism of intervention effects is essential in informing adaptions so that effectiveness of the intervention is not lost. The recent systematic review by McCrabb and colleagues highlights the decreased intervention effects when obesity interventions are scaled up – they found that effects on weight status, physical activity/sedentary behaviour, and nutrition reported in scaled-up interventions were typically 75% or less of the effects reported in pre–scale-up efficacy trials ^9^. Reporting of fidelity components in our review was also varied – training (95%), delivery (65%), and receipt (10%). This has been noted in other childhood obesity-related reviews ^80, 81^, and has important implications for the interpretation, as well as the generalisability, of study findings.

Despite calls for greater attention to external validity for almost 40 years now ^16, 82– 84^, we noted that problems with attention to generalisability persist. Only one trial within this review, the INSIGHT trial ^56^, reported on all elements of the external validity assessment tool developed by RL ^18, 32^. Earlier this year, Huebschmann and colleagues made a further call for increased attention to external validity ^82^. For trialists, there is a tension between internal validity and external validity, with preference historically for ensuring the former and minimising the risk of bias, at the expense of generalisability and applicability to real-world settings. Standard reporting guidelines such as the CONSORT statement for the reporting of randomized controlled trials ^85^, the CONSORT extension for cluster trials ^86^ and the CONSORT extension for pragmatic trials ^87^ traditionally focus on internal validity elements, with limited focus and guidance around external validity. The TIDieR reporting guidelines for intervention description and replication somewhat address this gap ^40^.

We acknowledge the challenging context in which triallists work and that there are many positive activities in this area. We have a number of suggestions for moving work in this area forward nevertheless. Triallists could plan their interventions with scalability and sustainability in mind, giving due consideration to the type of trial conducted as well as the intervention characteristics. Few researchers plan for the sustainability of their interventions ^88^. The aforementioned reporting guidelines can be used in combination to report on study findings, with additional materials published to enhance external validity assessment, including protocols and more detailed information made accessible via supplementary materials or open access repositories. Researchers could also use models such as RE-AIM to guide reporting of external validity elements. If researchers used RE-AIM as a planning tool when designing their intervention and evaluation, this might also overcome some of the difficulties in reporting on RE-AIM components relevant to external validity. Glasgow and Estabrooks note the challenges in comprehensively reporting on all RE-AIM dimensions within community and clinical settings with limited resources, however, highlighting that even well-funded NIH grants and published research studies, stating use of the RE-AIM framework, only employ it partially, and inconsistently when they do so ^89^. Inconsistencies in the degree to which authors report each RE-AIM dimension in its entirety as well as inaccuracies in reporting elements within each dimension have been highlighted by other authors also ^22, 90^. Further work is needed with researchers to embed such frameworks appropriately. As mentioned above, increasing the availability of protocols and more detailed information via supplementary materials or open access repositories is one such step. Research to understand the facilitators and barriers to reporting elements of external validity, as well as work with stakeholders to prioritise the most important elements/dimensions of external validity reporting would be useful to enhance work in this area. A recently published tool on assessment of scalability contains several elements relevant to external validity assessment ^91^. These include: costs and cost effectiveness, intervention characteristics, information on delivery agents, participation rate of settings, outcomes that can be compared to standards, effect moderators, adverse consequences, and acceptability. Such elements should be prioritised by researchers in planning studies and reporting findings.

Funding bodies, review panels, journals/journal editorial boards, and policymakers could also take action to promote the integration of external validity considerations into the funding, design, conduct, reporting, synthesis and translation of research ^19, 82, 84, 92^. This need not be at the expense of internal validity, and can help facilitate credible research and knowledge translation ^82, 92^. The inclusion of a PRECIS-2 graphic when proposing or reporting on a study can also be undertaken to enable the assessment of external validity ^82^.

### Strengths and limitations

The strengths of this work are the use of a comprehensive and rigorous methodology, including a broad search strategy and range of databases, no language restrictions, and the screening of trials and extraction of data by two independent review authors. A number of limitations, however, must be noted. While we included journal articles, protocols, grey literature and supplementary materials, it is possible that researchers of the reviewed studies may have collected some of the information required to complete the external validity assessment but did not report it in the articles published to date. Furthermore, the external validity tool only codes items as present, absent, or not applicable. The extent, or quality, to which the studies report on the various external validity elements, e.g. fidelity, is not assessed; this may result in an over-estimation of the reporting quality of some studies. While it is not necessary for all studies to be strong on all of the external validity criterion, researchers, decision-makers and others could use this information, if provided, to make judgments as to the applicability or generalisability of a study or review ^16^.

## Conclusion

This review examined the reporting of external validity elements within 39 studies encompassing 46 early-life health professional-delivered interventions. While such interventions have the potential to influence important health behaviours, in addition to child weight, we identified important gaps in the reporting of external validity elements within studies, and factors that could enhance translation and scale-up of interventions across all five external validity dimensions. External validity elements that were poorly described included: representativeness of individuals and settings, treatment receipt, intervention mechanisms and moderators, cost effectiveness, and intervention sustainability and acceptability. More emphasis is needed on research designs that consider generalisability, and the reporting of external validity elements in early life childhood obesity prevention interventions.

## Data availability

### Underlying data

All data underlying the results are available as part of the article and no additional source data are required.

### Extended data

Open Science Framework: Health professional-delivered obesity prevention interventions during the first 1,000 days: A systematic review of external validity reporting. https://doi.org/10.17605/OSF.IO/G2ZMY ^36^

This contains the following underlying data:

- SearchStrategy_v1_HealthProfessional-deliveredObesityPreventionF1D.pdf (Search strategy)
- DataExtractionForm_v3_HealthProfessional-deliveredObesityPreventionF1D.pdf (Data extraction form)
- InterventionDescriptions_v7_HealthProfessional-deliveredObesityPreventionF1D.xlsx (Intervention descriptions)
- InterventionOutcomes_v5_HealthProfessional-deliveredObesityPreventionF1D.xlsx (Intervention outcomes)
- FullDetailsofExternalValidityAssessments_v2_HealthProfessional-deliveredObesityPreventionF1D.pdf (Full details of external validity assessments)
- SummaryTable-ExternalValidity-ByStudy_v3_HealthProfessional-deliveredObesityPreventionF1D.xlsx (Summary table of external validity assessments by study)

### Reporting guidelines

Open Science Framework: PRISMA checklist for ‘Health professional-delivered obesity prevention interventions during the first 1,000 days: A systematic review of external validity reporting’. https://doi.org/10.17605/OSF.IO/G2ZMY ^36^

Data are available under the terms of the Creative Commons Zero "No rights reserved" data waiver (CC0 1.0 Public domain dedication).
