# Supplementary material for: Health professional-delivered obesity prevention interventions during the first 1,000 days: A systematic review of external validity reporting
Source: HRB Open Res. 2019 Oct 21;2:14. Originally published 2019 Jul 19. [Version 2] doi: 10.12688/hrbopenres.12924.2 (PMC6973534; doi:10.12688/hrbopenres.12924.2)
Supplement: Peer review history

# Peer review: Reviewer response for version 1

**DOI:** 10.21956/hrbopenres.14003.r26785

**Published:** 2019-09-16

**Authors:** Paula M. Watson

**Affiliations:** 1 Research Institute for Sport and Exercise Sciences, Liverpool John Moores University, Liverpool, UK

**Overview**

 This is a well-conducted systematic review that assesses the extent to which early life childhood obesity intervention studies report external validity components. Methods and results are clear and transparent, and the discussion is good with some appropriate recommendations for researchers.

 There is some ambiguity with terminology in places. As I understand it, the aim of this article is to assess the extent to which obesity prevention studies/trials *report* external validity components. However in places the authors refer to “evaluating external validity” and “evaluating external validity reporting” interchangeably. Whilst these processes are naturally linked, it needs to be clear they are distinct and the purpose of the article needs to be clearly outlined. I have highlighted some specific sections where this is unclear below, but it would be useful to check the whole article to ensure the aims are clear, and terminology is as intended throughout. 

 I also feel the introduction could benefit with some background information for non-specialist readers (see specific points below).

**Specific comments**

**Title/Throughout**

 It took me some time to understand what was meant by “the first 1000 days” (my initial interpretation was the first 1000 days of intervention implementation). For clarity, I think it would be useful to refer to “the first 1000 days of life” when using this phrase. It would also be useful to clarify for non-specialist readers that the first 1000 days of life includes the gestation period. Otherwise the inclusion criteria (which refers to 2 years, i.e. ~730 days) could appear to be misaligned with the aim/title.

**Abstract**

**Methods (3 ^rd^ line from bottom)** – would it be more accurate to say “Two authors independently assessed the external validity *reporting* of 20% of included studies”?

**Introduction**

 For the non-specialist reader, it might be useful to insert a paragraph at the start of the introduction to outline the current state of play in early childhood obesity prevention, e.g. explain current obesity prevalence when starting school, highlight the importance of obesity prevention interventions within infancy (and during pregnancy), and outline what these interventions typically involve. 

**3 ^rd ^paragraph, lines 14-15** – suggest re-wording the final part of this sentence for accuracy. Whilst the RE-AIM model might provide a framework for *evaluating external validity reporting*, this was not the primary reason it was developed. In my understanding, the purpose of RE-AIM is to guide researchers in the planning, evaluation and reporting of interventions to maximise external validity (as correctly stated in the first part of the sentence, lines 10-13). 

**Methods**

**Data extraction paragraph, lines 9-13 **– the authors indicate that the external validity assessment tool was used “to assess the extent to which findings of included studies could be generalised to populations or settings beyond those in the original study”. This implies that the data being extracted was about the *external validity* of interventions, rather than the *reporting *of external validity of interventions? Is this correct? Please clarify and amend wording as appropriate.

**Data extraction, lines 15-16 **– as above, shouldn’t this read external validity *reporting*?

**Results**

**Paragraph 3, line 1** – the description of Table 2 suggests the table includes an assessment of the external validity of studies. This is a little misleading - the table reports on the extent to which external validity aspects are *reported*, but does not include an external validity assessment of the studies themselves. Suggest re-wording to make this clear.

**Figure 1 (Prisma flow diagram)** – in the “Full text articles excluded, with reasons” box, it would be useful to replace the word “wrong” with details of *why* the intervention/population/study design did not fit. E.g. “Intervention did not do XXX”, or “Study had no comparator group”. This would be more informative for readers. 

**Discussion/Conclusion**

**Paragraph 1, lines 3-5** – “an understanding of the external validity of such interventions is vital”. It is important to make clear this paper makes no attempt to improve our understanding of the external validity of interventions, the aim is to understand the extent to which process elements are reported (as reporting is crucial so that others can assess the external validity of interventions). 

**Paragraph 3, final line** – it is worth being mindful that the TIDieR guidelines only address some aspects of “external validity”. Other elements such as reach and representativeness, adoption, and maintenance (settings) might require researchers to draw on RE-AIM also.

**Paragraph 4** – as RE-AIM is intended as a planning and evaluation tool, it would be useful to integrate RE-AIM into the discussion around planning for sustainability of interventions. If researchers use RE-AIM as a planning tool when designing their intervention and evaluation, this might also overcome some of the difficulties in reporting on RE-AIM components (as discussed later in the paragraph).

I confirm that I have read this submission and believe that I have an appropriate level of expertise to confirm that it is of an acceptable scientific standard.

# Response

**Published:** 2019-10-21

**Authors:** Marita Hennessy

**Affiliations:** NUI Galway, Ireland

This is a well-conducted systematic review that assesses the extent to which early life childhood obesity intervention studies report external validity components. Methods and results are clear and transparent, and the discussion is good with some appropriate recommendations for researchers.

**Reviewer Comment 1:** There is some ambiguity with terminology in places. As I understand it, the aim of this article is to assess the extent to which obesity prevention studies/trials report external validity components. However in places the authors refer to “evaluating external validity” and “evaluating external validity reporting” interchangeably. Whilst these processes are naturally linked, it needs to be clear they are distinct and the purpose of the article needs to be clearly outlined. I have highlighted some specific sections where this is unclear below, but it would be useful to check the whole article to ensure the aims are clear, and terminology is as intended throughout.

***Author Response 1: ***The manuscript has been reviewed and terminology clarified to reflect the focus on “evaluating external validity reporting”.

I also feel the introduction could benefit with some background information for non-specialist readers (see specific points below).

Specific comments

Title/Throughout

**Reviewer Comment 2: **It took me some time to understand what was meant by “the first 1000 days” (my initial interpretation was the first 1000 days of intervention implementation). For clarity, I think it would be useful to refer to “the first 1000 days of life” when using this phrase. It would also be useful to clarify for non-specialist readers that the first 1000 days of life includes the gestation period. Otherwise the inclusion criteria (which refers to 2 years, i.e. ~730 days) could appear to be misaligned with the aim/title.

***Author Response 2:*** We have defined what we mean by first 1,000 days in the abstract and introduction.

Abstract

**Reviewer Comment 3:** Methods (3 line from bottom) – would it be more accurate to say “Two authors independently assessed the external validity reporting of 20% of included studies”?

***Author Response 3:*** This sentence now reads “Two authors independently assessed the external validity reporting of 20% of included studies; discrepancies were resolved, and then one author completed assessments of the remaining studies.”.

Introduction

**Reviewer Comment 4:** For the non-specialist reader, it might be useful to insert a paragraph at the start of the introduction to outline the current state of play in early childhood obesity prevention, e.g. explain current obesity prevalence when starting school, highlight the importance of obesity prevention interventions within infancy (and during pregnancy), and outline what these interventions typically involve.

***Author Response 4:*** We have somewhat addressed this issue in the introduction: “Health professional-delivered interventions to prevent childhood obesity during the first 1,000 days of life (i.e. the period from conception to a child’s second birthday) have limited impacts on adiposity/weight outcomes, but have more positive impacts on behavioural outcomes ^36^. The paper quoted in this section provides further details on the current state of play in early life obesity prevention. The main focus of the current paper is on the reporting of external validity elements within interventions. That said, we have added the following to the introduction: “In 2018, approximately 41 million children under the age of five years were classified as having overweight or obesity ^1^. Child obesity prevention is a public health priority, with early life intervention advocated ^2^. Effective, scalable, and affordable strategies that do not widen health inequities are needed to address this issue ^3, 4^.”

**Reviewer Comment 5:** 3 paragraph, lines 14-15 – suggest re-wording the final part of this sentence for accuracy. Whilst the RE-AIM model might provide a framework for evaluating external validity reporting, this was not the primary reason it was developed. In my understanding, the purpose of RE-AIM is to guide researchers in the planning, evaluation and reporting of interventions to maximise external validity (as correctly stated in the first part of the sentence, lines 10-13).

***Author Response 5:*** This sentence now reads: “To improve reporting across behavioural interventions and enhance the translation of research into practice, Glasgow and colleagues developed the RE-AIM (Reach, Effectiveness, Adoption, Implementation, Maintenance) framework ^19,20^.”

Methods

**Reviewer Comment 6: **Data extraction paragraph, lines 9-13 – the authors indicate that the external validity assessment tool was used “to assess the extent to which findings of included studies could be generalised to populations or settings beyond those in the original study”. This implies that the data being extracted was about the external validity of interventions, rather than the reporting of external validity of interventions? Is this correct? Please clarify and amend wording as appropriate.

***Author Response 6:*** This sentence now reads: “The external validity assessment tool previously developed by RL ^18,32^ was used to assess the extent to which included studies/trials reported on elements that would aid decision-making  around whether the findings of such studies/trials could be generalised to populations or settings beyond those in the original study ^21^.”

**Reviewer Comment 7:** Data extraction, lines 15-16 – as above, shouldn’t this read external validity reporting?

***Author Response 7: ***This sentence now reads “Initially, two authors (MH and RL) independently assessed the external validity reporting of 20% of included studies.”.

Results

**Reviewer Comment 8:** Paragraph 3, line 1 – the description of Table 2 suggests the table includes an assessment of the external validity of studies. This is a little misleading - the table reports on the extent to which external validity aspects are reported, but does not include an external validity assessment of the studies themselves. Suggest re-wording to make this clear.

***Author Response 8:*** This now reads: “The assessment of the reporting of external validity elements of the 39 included studies is summarised in Table 2, with a summary by study available as extended data ^36 ^.”

**Reviewer Comment 9:** Figure 1 (Prisma flow diagram) – in the “Full text articles excluded, with reasons” box, it would be useful to replace the word “wrong” with details of why the intervention/population/study design did not fit. E.g. “Intervention did not do XXX”, or “Study had no comparator group”. This would be more informative for readers.

***Author Response 9:*** We have reported reasons for exclusion in line with Cochrane guidelines. Adding additional information would not add more detail than that provided in the PRISMA diagram and in the inclusion criteria outlined in Table 1, e.g. wrong intervention = not a behavioural intervention designed to prevent obesity (by directly/explicitly focusing on childhood obesity prevention, or by indirectly focusing on childhood obesity-related risk factors); wrong outcomes = no child weight/anthropometric outcomes reported; wrong study design = not a RCT or quasi-RCT.

Discussion/Conclusion

**Reviewer Comment 10:** Paragraph 1, lines 3-5 – “an understanding of the external validity of such interventions is vital”. It is important to make clear this paper makes no attempt to improve our understanding of the external validity of interventions, the aim is to understand the extent to which process elements are reported (as reporting is crucial so that others can assess the external validity of interventions).

***Author Response 10:*** This section now reads: “Early life interventions delivered by health professionals have the potential to influence important health behaviours, in addition to child weight. Understanding the reporting of external validity elements of such interventions is vital to address their potential for translation and scalability, as well as replication efforts. In this systematic review we identified 39 studies, representing 46 interventions. External validity elements that were generally well reported included target populations and settings, participant inclusion and exclusion criteria, intervention characteristics, delivery agents, outcomes, and attrition. Similar to other reviews of childhood obesity interventions ^17, 18 ^, however, we identified important gaps in the reporting of external validity elements within studies, and factors that could enhance translation and scale-up of interventions across all five external validity dimensions. External validity elements that were poorly reported included: representativeness of individuals and settings, treatment receipt, intervention mechanisms and moderators, cost effectiveness, and intervention sustainability and acceptability.”

**Reviewer Comment 11:** Paragraph 3, final line – it is worth being mindful that the TIDieR guidelines only address some aspects of “external validity”. Other elements such as reach and representativeness, adoption, and maintenance (settings) might require researchers to draw on RE-AIM also.

***Author Response 11: ***We agree – this is a point that we pick up on in the following paragraph: “The aforementioned reporting guidelines can be used in combination to report on study findings, with additional materials published to enhance external validity assessment, including protocols and more detailed information made accessible via supplementary materials or open access repositories. Researchers could also use models such as RE-AIM to guide reporting of external validity elements.”

**Reviewer Comment 12:** Paragraph 4 – as RE-AIM is intended as a planning and evaluation tool, it would be useful to integrate RE-AIM into the discussion around planning for sustainability of interventions. If researchers use RE-AIM as a planning tool when designing their intervention and evaluation, this might also overcome some of the difficulties in reporting on RE-AIM components (as discussed later in the paragraph).

***Author Response 12: ***We have added the following sentence to this paragraph: “If researchers used RE-AIM as a planning tool when designing their intervention and evaluation, this might also overcome some of the difficulties in reporting on RE-AIM components relevant to external validity.”.

# Peer review: Reviewer response for version 1

**DOI:** 10.21956/hrbopenres.14003.r26701

**Published:** 2019-08-29

**Authors:** Luke Wolfenden

**Affiliations:** 1 Hunter New England Population Health, Wallsend, Australia

This systematic review examined the extent to which childhood obesity interventions delivered by health professionals during the first 1,000 days report on information to enable assessment of the generalisability of trial findings. Generalisability is an important attribute of public health intervention trials. While a number of studies have described reporting of trial information that may facilitate assessments of trial generalizability, there does not appear to have been previous work in this area on obesity in this important population group. The manuscript is well written, and the study methods are appropriate. I congratulate the authors on well undertaken review. I provide below points for clarification and suggestions for further improvement.

1. It may be helpful to provide an early definition of external validity, and trial generalisability in the introduction to set the scene. These terms are not necessarily inter-changeable but at times it appears as though they are used that way.
2. The introduction risks conflating external validity with scalability (among other things). It would help to provide a more nuanced description of how external validity is related to processes of scaling up, and also other processes suggested by the authors including replication and translation. A study (and its characteristics/findings) may not be particularly generalizable, but still meet local end-user decision making needs (particularly if it was specifically designed for or conducted with them). In the review the authors state that “An understanding of the external validity of such interventions is vital to address their potential for translation and scalability, as well as replication efforts” – I think what I am looking for is some text in the introduction stating how and why that’s the case.
3. The manuscript presents a large list of factors important in assessment of trial generalisability. I wondered if there was any evidence regarding which of this comprehensive list, were most important/useful. The authors employed comprehensive systematic review methods in the identification of trials, including searching multiple data-bases, prospective registration and duplicate screening. While not expressed in as much detail in this manuscript, the authors provide references where such detail is available.
4. The external validity assessment tool used also appears comprehensive and appropriate for the study objectives. While the items in the tool are described in the tables, it would be nice if the authors could characterise the tool a little more in the methods text, to summarise the information it seeks to extract.
5. Data were extracted by one author (MH) using a pre-piloted data extraction tool (see extended data ^33^), with 20% double-checked by a second reviewer. Are the authors able to report the inter-rater agreement where duplicate extraction was undertaken.
6. The results are clearly presented in table and text form.
7. The discussion does a good job of summarising and interpreting the key findings of the review, and appropriately acknowledges the study limitations.
8. As the authors state, the focus of the majority of studies was on establishing efficacy rather than effectiveness or how such interventions could be scaled up and translated into routine practice. I did wonder if efficacy trials which are explicitly and purposefully undertaken in ideal research conditions, and so are not designed with the intent of providing knowledge generalizable to routine clinical settings, should be held to the same standard of such reporting as studies (e.g effectiveness trials) which are intended to provide more externally valid findings. Perhaps this is worth discussing.

Similarly, at first glance the external validity tool used in this study appears a high bench mark for researchers to achieve given existing demands on research participants for data collection, word restrictions in peer reviewed journals, and research funding limits. I'm not discounting the importance of reporting information to allow an assessment of generalisability, but can you offer some recommendations as to how researchers can best manage this?

I confirm that I have read this submission and believe that I have an appropriate level of expertise to confirm that it is of an acceptable scientific standard.

# Response

**Published:** 2019-10-21

**Authors:** Marita Hennessy

**Affiliations:** NUI Galway, Ireland

This systematic review examined the extent to which childhood obesity interventions delivered by health professionals during the first 1,000 days report on information to enable assessment of the generalisability of trial findings. Generalisability is an important attribute of public health intervention trials. While a number of studies have described reporting of trial information that may facilitate assessments of trial generalizability, there does not appear to have been previous work in this area on obesity in this important population group. The manuscript is well written, and the study methods are appropriate. I congratulate the authors on well undertaken review. I provide below points for clarification and suggestions for further improvement.

**Reviewer Comment 1:** It may be helpful to provide an early definition of external validity, and trial generalisability in the introduction to set the scene. These terms are not necessarily inter-changeable but at times it appears as though they are used that way. The introduction risks conflating external validity with scalability (among other things). It would help to provide a more nuanced description of how external validity is related to processes of scaling up, and also other processes suggested by the authors including replication and translation. A study (and its characteristics/findings) may not be particularly generalizable, but still meet local end-user decision making needs (particularly if it was specifically designed for or conducted with them). In the review the authors state that “An understanding of the external validity of such interventions is vital to address their potential for translation and scalability, as well as replication efforts” – I think what I am looking for is some text in the introduction stating how and why that’s the case.

***Author Response 1:*** We have updated the first paragraph of the introduction to read: “Appraising scalability prior to investment is vital ^8^ ^, 9 ^. Scalability is defined by the World Health Organization as ‘the deliberate effort to increase the impact of successfully tested health interventions so as to benefit more people and to foster policy and program development on a lasting basis’ ^10^. An understanding of the external validity of the intervention is critical to determining scalability, in addition to understanding the fit between an intervention and the political and strategic context. External validity refers to the generalizability of the results of an intervention to or across target populations or settings, while applicability refers to generalizability to any populations or settings ^11^. To understand the external validity of an intervention, decision makers need to have sufficient information on the reach and acceptability of the intervention, core intervention components required for fidelity, any differential effects on the target population, unintended consequences, costs versus benefits, and the clinical or policy significance of the intervention effects to inform decisions about whether interventions should be scaled-up ^12^^– ^^16^. The poor reporting of external validity elements in childhood obesity prevention research also limits decision-makers’ ability to determine the likely scalability of these interventions, limiting the implementation of effective interventions into routine practice ^17^^, ^^18^ ."

**Reviewer Comment 2: **The manuscript presents a large list of factors important in assessment of trial generalisability. I wondered if there was any evidence regarding which of this comprehensive list, were most important/useful. The authors employed comprehensive systematic review methods in the identification of trials, including searching multiple data-bases, prospective registration and duplicate screening. While not expressed in as much detail in this manuscript, the authors provide references where such detail is available.

***Author Response 2: ***We are not aware of any evidence. That said, we have included the following in the discussion: “A recently published tool on assessment of scalability contains several elements relevant to external validity assessment ^91^. These include: costs and cost effectiveness, intervention characteristics, information on delivery agents, participation rate of settings, outcomes that can be compared to standards, effect moderators, adverse consequences, and acceptability. Such elements should be prioritised by researchers in planning studies and reporting findings.”

**Reviewer Comment 3:** The external validity assessment tool used also appears comprehensive and appropriate for the study objectives. While the items in the tool are described in the tables, it would be nice if the authors could characterise the tool a little more in the methods text, to summarise the information it seeks to extract.

***Author Response 3:*** We have moved the following information about the tool from the introduction to the methods section: “This adapted tool includes five main dimensions (defined in Table 2): 1) reach and representativeness (individuals); 2) reach and representativeness (settings); 3) implementation and adaptation (of intervention), which includes fidelity considerations; 4) outcomes for decision makers; 5) maintenance and institutionalisation (i.e. the potential for implementation of the intervention in routine service delivery)”. We felt it would be too repetitive to include it in the methods text also.

**Reviewer Comment 4:** Data were extracted by one author (MH) using a pre-piloted data extraction tool (see extended data ), with 20% double-checked by a second reviewer. Are the authors able to report the inter-rater agreement where duplicate extraction was undertaken.

***Author Response 4:*** We have added the following statement to the results section: “Inter-rater reliability, using percent agreement, was high (88.2%).”

**Reviewer Comment 5: **The results are clearly presented in table and text form.

***Author Response 5:*** Thank you.

**Reviewer Comment 6: **The discussion does a good job of summarising and interpreting the key findings of the review, and appropriately acknowledges the study limitations.

***Author Response 6: ***Thank you.

**Reviewer Comment 7: **As the authors state, the focus of the majority of studies was on establishing efficacy rather than effectiveness or how such interventions could be scaled up and translated into routine practice. I did wonder if efficacy trials which are explicitly and purposefully undertaken in ideal research conditions, and so are not designed with the intent of providing knowledge generalizable to routine clinical settings, should be held to the same standard of such reporting as studies (e.g. effectiveness trials) which are intended to provide more externally valid findings. Perhaps this is worth discussing.

***Author Response 7:*** We have added the following to this section: “It could be argued that efficacy trials should not be held to the same level of accountability regarding reporting of external validity elements. Such information however is important regardless of trial type, not only to inform generalisability, but also to enhance understanding of the active ingredients of interventions and core components to retain in effectiveness trials or the scale-up of interventions.”

**Reviewer Comment 8**: Similarly, at first glance the external validity tool used in this study appears a high bench mark for researchers to achieve given existing demands on research participants for data collection, word restrictions in peer reviewed journals, and research funding limits. I'm not discounting the importance of reporting information to allow an assessment of generalisability, but can you offer some recommendations as to how researchers can best manage this?

***Author Response 8:*** We have added the follow to the discussion: “As mentioned above, increasing the availability of protocols and more detailed information via supplementary materials or open access repositories is one such step. Research to understand the facilitators and barriers to reporting elements of external validity, as well as work with stakeholders to prioritise the most important elements/dimensions of external validity reporting would be useful to enhance work in this area.”
